# Type II NKT Cells Stimulate Diet-Induced Obesity by Mediating Adipose Tissue Inflammation, Steatohepatitis and Insulin Resistance

**DOI:** 10.1371/journal.pone.0030568

**Published:** 2012-02-22

**Authors:** Masashi Satoh, Yasuhiro Andoh, Christopher Stuart Clingan, Hisako Ogura, Satoshi Fujii, Koji Eshima, Toshinori Nakayama, Masaru Taniguchi, Noriyuki Hirata, Naoki Ishimori, Hiroyuki Tsutsui, Kazunori Onoé, Kazuya Iwabuchi

**Affiliations:** 1 Department of Immunology, Kitasato University School of Medicine, Sagamihar, Japan; 2 Division of Immunobiology, Research Section of Pathophysiology, Institute for Genetic Medicine, Hokkaido University, Sapporo, Japan; 3 Department of Cardiovascular Medicine, Hokkaido University Graduate School of Medicine, Sapporo, Japan; 4 Department of Microbiology and Immunology, Vanderbilt University School of Medicine, Nashville, Tennessee, United States of America; 5 Department of Molecular and Cellular Pathobiology and Therapeutics, Graduate School of Pharmaceutical Sciences, Nagoya City University, Nagoya, Japan; 6 Department of Immunology, Graduate School of Medicine, Chiba University, Chiba, Japan; 7 RIKEN Research Center for Allergy and Immunology, Yokohama, Japan; 8 Division of Cancer Biology, Research Section of Pathophysiology, Institute for Genetic Medicine, Hokkaido University, Sapporo, Japan; University of Birmingham, United Kingdom

## Abstract

The progression of obesity is accompanied by a chronic inflammatory process that involves both innate and acquired immunity. Natural killer T (NKT) cells recognize lipid antigens and are also distributed in adipose tissue. To examine the involvement of NKT cells in the development of obesity, C57BL/6 mice (wild type; WT), and two NKT-cell-deficient strains, Jα18^−/−^ mice that lack the type I subset and CD1d^−/−^ mice that lack both the type I and II subsets, were fed a high fat diet (HFD). CD1d^−/−^ mice gained the least body weight with the least weight in perigonadal and brown adipose tissue as well as in the liver, compared to WT or Jα18^−/−^ mice fed an HFD. Histologically, CD1d^−/−^ mice had significantly smaller adipocytes and developed significantly milder hepatosteatosis than WT or Jα18^−/−^ mice. The number of NK1.1^+^TCRβ^+^ cells in adipose tissue increased when WT mice were fed an HFD and were mostly invariant Vα14Jα18-negative. CD11b^+^ macrophages (Mφ) were another major subset of cells in adipose tissue infiltrates, and they were divided into F4/80^high^ and F4/80^low^ cells. The F4/80^low^-Mφ subset in adipose tissue was increased in CD1d^−/−^ mice, and this population likely played an anti-inflammatory role. Glucose intolerance and insulin resistance in CD1d^−/−^ mice were not aggravated as in WT or Jα18^−/−^ mice fed an HFD, likely due to a lower grade of inflammation and adiposity. Collectively, our findings provide evidence that type II NKT cells initiate inflammation in the liver and adipose tissue and exacerbate the course of obesity that leads to insulin resistance.

## Introduction

Obesity is thought to progress with caloric excess under a chronic inflammatory process characterized by infiltration of adipose tissue by Mφ and by cells of the adaptive immune system, such as T cells [1]–[3]. The inflammation in adipose tissue induces alterations in metabolic and endocrine functions of adipocytes, which leads to insulin resistance and various pathological responses [4], [5]. Recent studies by Nishimura et al revealed the active participation of CD8^+^ T cells in chronic inflammation in adipose tissue [6]. Moreover, CD4^+^Foxp3^+^ T cells with unique specificity have been detected in adipose tissue and were suggested to regulate the development of obesity by suppressing inflammatory responses [7]. Furthermore, additional findings showed that the transfer of CD4^+^ T cells from WT but not from T-cell receptor transgenic mice ameliorated the metabolic dysregulation in Rag-1^−/−^ mice fed a high fat diet (HFD), which led to the idea that CD4^+^ T cells play a suppressive role in diet-induced obesity (DIO) [8]. These studies have indicated that T cells that infiltrate adipose tissue are not just inert bystanders but are active modifiers of inflammation and thus either aggravate or ameliorate obesity.

Natural killer T (NKT) cells are a unique subset of T-lineage cells that recognize various lipid antigens in the context of CD1d molecules [9]. Among lipid ligands, α-galactosylceramide (α-GalCer) is the prototype ligand [10] that can stimulate NKT cells to promptly produce large amounts of various cytokines and chemokines and also demonstrate cytocidal activity [11]. Endogenous ligands can also stimulate NKT cells to perform their innate effector functions [12]. Moreover, NKT cells localize to the liver [13], where lipid metabolism is active, and in adipose tissue [14], another location for lipid metabolism with endocrine functions. These considerations led us to suggest that, NKT cells might play a role in a disease that involves abnormal lipid metabolism or lipid-related inflammation. Indeed, several research groups including our team have demonstrated that NKT cells accelerate atherogenesis in a mouse model of atherosclerosis [15]–[17]. Furthermore, we have examined the involvement of NKT cells in insulin resistance induced in mice fed an HFD and demonstrated that NKT cells play an important role in adipose-tissue inflammation and glucose intolerance in β_2_-microglobulin knockout (β_2_m^−/−^) mice with DIO [18]. However, both mainstream CD8^+^ T cells and various innate lymphocytes other than NKT cells also are absent in β_2_m^−/−^ mice [19]. Thus, we attempted to examine the involvement of NKT cells in DIO and insulin resistance using NKT cell-deficient mice. To this end, we compared B6 (WT) and two strains of NKT cell-deficient mice, CD1d^−/−^ and Jα18^−/−^ mice on a B6 genetic background. Unlike our previous study in β_2_m^−/−^ mice [18], DIO was significantly suppressed in CD1d^−/−^ mice compared to WT mice. Moreover, in Jα18^−/−^ mice where type I but not type II NKT cells were deficient, DIO was induced to an equal extent as in WT mice. The possible mechanisms underlying lipid-induced NKT-cell activation and the development of chronic inflammation by type II NKT cells in DIO are discussed.

## Materials and Methods

### Mice

Male and female 8-week-old C57BL/6 (B6; Nippon SLC, Shizuoka, Japan), B6.CD1d^−/−^ [20], and B6.Jα18^−/−^ [21] mice were used. Mice were maintained on food and water *ad libitum* until they reached the desired weight (20–24 g) or age (8 wk) under specific pathogen-free conditions. All experiments were approved by the Committees of Animal Experimentation at Hokkaido University (permit number: #09-0022) and Kitasato University (permit number: #2011 105).

### Diet-induced obesity

Mice were fed either regular chow as a standard fat diet (SFD; Nihon Nosan: fat 4.3%, cholesterol 0.03%, protein 18.3%, carbohydrate 58.3%) or an HFD (CLEA Japan HFD-32: fat (powdered tallow and safflower oil of high oleic type) 32.0%, protein 25.5%, fiber 2.9%, mineral 4.0%, nitrogen 29.4%, water 6.2%) starting from 8 wk of age for 18 wk. Mice were weighed weekly. The ingredients of HFD-32 are listed in detail at <http://www.clea-japan.com/Feed/pdf/clea_hfd32.pdf>. Major fatty acids included oleic acid (C_18∶1_; 64.3%), palmitic acid (C_16∶0_; 12.6%), linoleic acid (C_18∶2_; 10.2%), and stearic acid (C_18∶0_; 7.5%). After 18 wk of feeding, mice were sacrificed for analysis. For some experiments, mice were injected intraperitoneally with α-GalCer (0.1 µg/g body weight, BW) or vehicle as control to examine the change of BW.

### Blood chemistry

Total cholesterol (T-chol), high-density lipoprotein (HDL) cholesterol (HDL-chol), triglyceride (TG), and alanine aminotransferase (ALT) concentrations in sera were quantified by colorimetric assays with the Fuji Drychem system (Fujifilm Medical, Osaka, Japan) according to the manufacturer's protocol, as described elsewhere [16].

### Histology and quantitative analyses of microscopic images

Perigonadal fat tissue was removed and fixed with buffered formaldehyde solution (10%) followed by ordinary processing for paraffin-embedded sections and hematoxylin-eosin (HE) staining. Images of the HE-stained adipose tissue were incorporated with a BIOREVO microscope (BZ8100; Keyence Corp., Osaka, Japan), and morphometric analyses were performed with image analysis software (BZ-H2A, -H1C) equipped on the microscope. Liver samples were snap-frozen in OCT compound (Sakura Finetek Co., Tokyo, Japan) with liquid nitrogen, and cryosections were stained with Oil Red O (ORO) (Sigma Chemical Co., St. Louis, MO) and Meyer's hematoxylin (Wako Pure Chemical Co. Ltd., Osaka, Japan). Images of lipid droplets in hepatocytes stained red were quantified by computerized image analysis system (Scion Image software, Scion Corp., Frederick, MD).

### Flow cytometry

Splenocytes were prepared by mincing the spleen with a glass homogenizer, and red blood cells were lysed with Tris-NH_4_Cl solution. Hepatic mononuclear cells (HMNC) were isolated from liver homogenates by density-gradient centrifugation with 33% Percoll™ (GE Healthcare Life Sciences, Piscataway, NJ) as previously reported [22]. Stromal vascular cells were isolated from the digest of perigonadal fat by mincing and incubating with collagenase D solution (2 mg/ml) (Roche Diagnostics, Indianapolis, IN) for 1–1.5 h. The cells were incubated with 2.4G2 monoclonal antibody (mAb) (anti-FcγRIII/II) to block non-specific binding of primary mAb and then reacted with CD1d:Ig recombinant fusion protein (BD Biosciences Pharmingen, San Diego, CA) loaded with α-GalCer (α-GalCer-CD1d-dimer), followed by detection with phycoerythrin (PE)-conjugated anti-mouse IgG_1_ mAb (A85-1; BD) according to the manufacturer's protocol [16]. After washing, cells were stained with a combination of the following mAb conjugates: fluorescein isothiocyanate (FITC)-anti-TCRβ chain (H57-597; BD), -anti-CD3ε (145-2C11; BD), -anti-CD1d (1B1; BD), -anti-CD206 (MR5D3; BioLegend, San Diego, CA), -rat IgG_2a_ (BD), -rat IgG_2b_ (BD), allophycocyanin (APC)-anti-NK1.1 (PK136; BD, BioLegend), -anti-CD11c (HL3; BD), -Streptavidin (BioLegend), PE-anti-CD4 (RM4-5; BD), -anti-CD8 (53-6.7; BD), -anti-CD11b (M1/70; BioLegend), and APC-Cy7-Streptavidin (BD). Stained cells were acquired with FACS Calibur or Canto II flow cytometers (BD Bioscience Immunocytometry Systems, San Jose, CA) and analyzed with CellQuest, FACS Diva (BDIS), or FlowJo (Tommy Digital Biology, Tokyo, Japan) software as described elsewhere [16]. Propidium iodide (PI; Sigma) or 7-aminoactinomycin D (7-AAD; BD) positive cells were electronically gated as dead cells from the analysis.

### Quantification of serum cytokines and adipokines

The concentration of Th1/Th2 and inflammatory cytokines, including IFN-γ, tumor necrosis factor (TNF)-α, IL-1α, 2, 4, 5, 6, 10, 17, and GM-CSF in serum was quantified with Mouse Th1/Th2 10plex FlowCytomix™ Multiplex (Bender MedSystems GmbH, Vienna, Austria) according to the manufacturer's protocol with a flow cytometer. Leptin (Morinaga Institute of Biological Sciences, Kanagawa, Japan) in sera was respectively quantified with enzyme-linked immunosorbent assay (ELISA) kit according to the manufacturers' protocol.

### IPGTT and insulin tolerance test

Intraperitoneal (*i.p.*) glucose tolerance test (IPGTT) was performed by *i.p.* injection of glucose solution (1 g/kg) after 16 h of fasting [6]. The insulin tolerance test (ITT) was performed by *i.p.* injection of insulin (0.751U/kg; Humulin R 100 U/ml; Eli Lilly Japan KK, Kobe, Japan) after 3.5 h of fasting. The blood glucose level was serially quantified with a blood glucose monitor (MEDISAFE MINI; Terumo Corp., Tokyo, Japan). The insulin level was also quantified with the mouse insulin ELISA kit (Morinaga) according to the manufacturer's protocol.

### HMNC transfer

HMNC were obtained from either Jα18^−/−^ or CD1d^−/−^ mice and were intravenously transferred to CD1d^−/−^ recipient mice (1×10^6^/mouse) at 8 wk. These mice were fed an HFD for 14 weeks and tested for IPGTT and ITT as above.

### Culture of Mϕ from the stromal vascular fraction (SVF) of adipose tissue

Cells (1×10^5^/well) obtained from perigonadal adipose tissue were cultured in RPMI medium (supplemented with 10% heat-inactivated fetal calf serum, 100 U/ml penicillin, 100 µg/ml streptomycin, and 50 µM β-mercaptoethanol) with 1 µg/ml LPS for 20 h. After 20 h, supernatants were collected and frozen at −80°C, and cytokines were later quantified as described above.

### Statistical analysis

Results were demonstrated as means ± standard deviation (s.d.). Statistical analysis between two groups was performed by Student's *t*-test and among three groups was performed using ANOVA followed by Tukey-Kramer tests. Pearson's correlation coefficient test was used to examine the correlation. Values with *P<0.05* were considered statistically significant.

## Results

### Impact of the presence of NKT cells on the weight gain of mice fed an HFD

**Figure 1:**
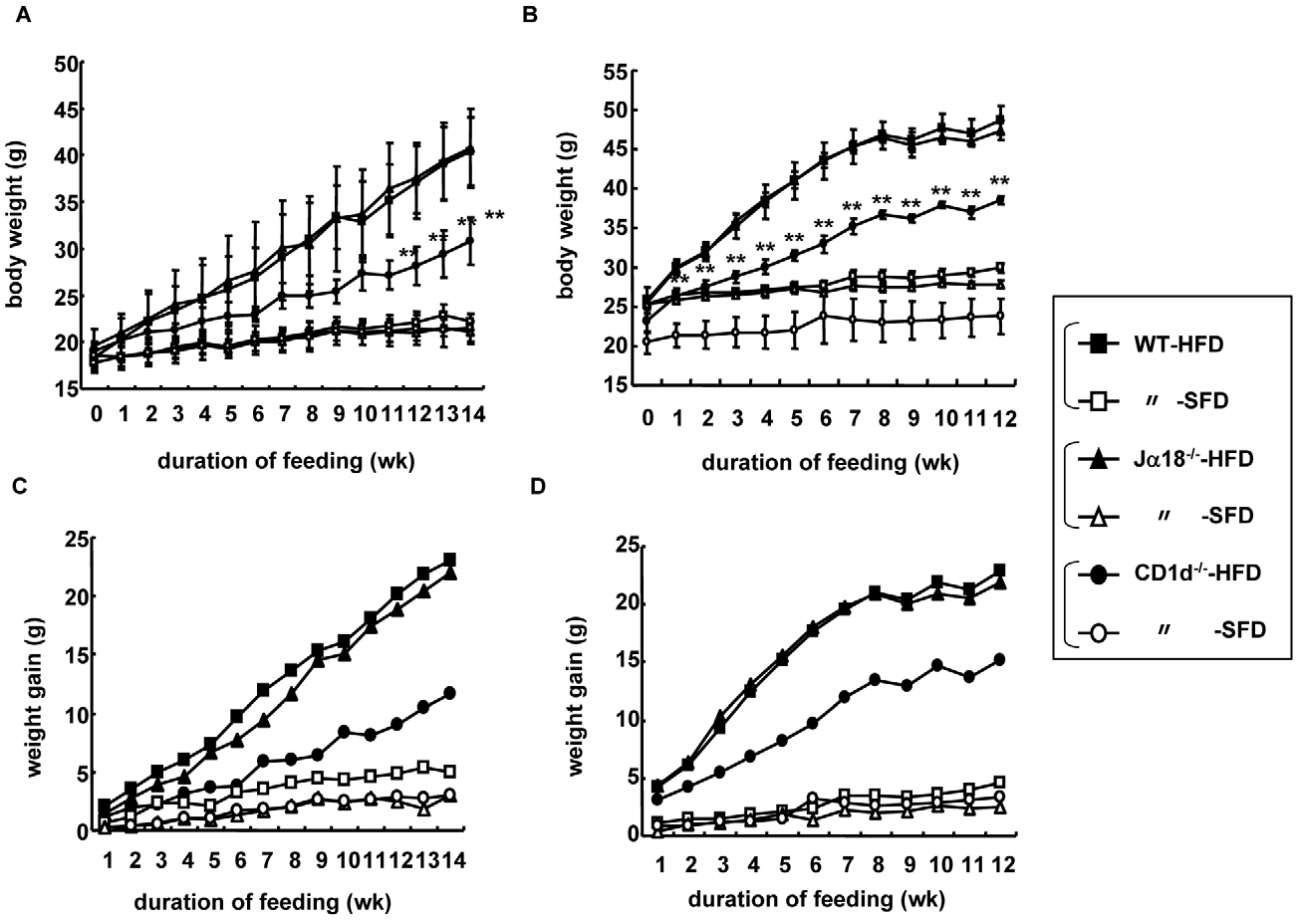
Body weight and weight gain of three strains of mice fed an SFD or an HFD. (A, B) Body weight (BW) of female (A) and male mice (B) fed a high-fat diet (HFD) or a standard-fat diet (SFD) at 8 wk and weighed weekly. (n = 3–7 in each group) (C, D) Representative data of three similar experiments are shown. Weight gain (ΔBW = BW_n wk_−BW_8 wk_; n≥9) of female (C) and male (D) mice. Each point represents mean ± standard deviation (s.d.). Statistical analysis was performed according to the Tukey-Kramer test. **p<0.01 (WT and Jα18^−/−^ vs CD1d^−/−^).

Female or male B6 (WT), B6.Jα18^−/−^ (Jα18^−/−^), and B6.CD1d^−/−^ (CD1d^−/−^) mice were fed either an SFD or an HFD from 8 wk to 22∼26 wk of age (for 14∼18 wk), and the BW of each group of mice was plotted over time. Three groups of female (Figure 1A) or male (Figure 1B) mice showed a similar and gradual increase of BW when fed an SFD. On the other hand, all groups of female and male mice gained substantially more BW on HFD than those on SFD. However, CD1d^−/−^ mice showed significantly less gains than those of WT and Jα18^−/−^ mice (Figure 1A, B). Consistently, the least difference was observed in CD1d^−/−^ mice when the net gain ([BW on HFD at *n* wk]−[BW on HFD at 8 wk]: n≥8 wk) was compared (Figure 1C, D). We also compared BW in WT mice that received either the prototypical type I NKT cell ligand α-GalCer or vehicle when fed either an SFD or an HFD. There was no significant difference in weight gain between mice that received α-GalCer or vehicle and fed either an SFD or an HFD (Figure S1A), suggesting that type I NKT cells might have a minimal role in the development of DIO even when the activating ligand is provided.

**Figure 2:**
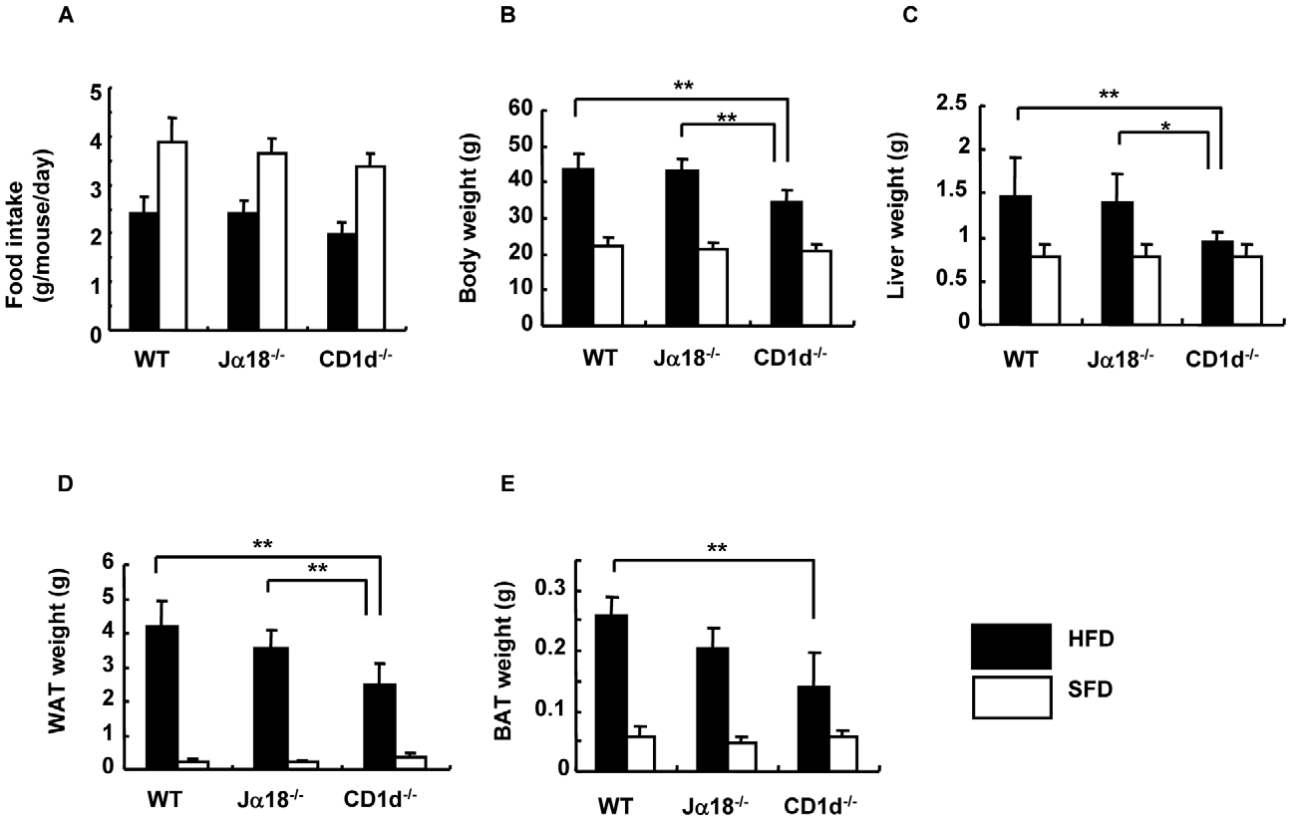
Physiological parameters of three strains of female mice fed an SFD or an HFD. (A) Food intake of mice in each group (g/mouse/day). (B–E) The body, liver, perigonadal adipose tissue (WAT), and brown adipose tissue (BAT) were weighed after 18 wk of feeding at 26 wk of age (n = 3–7 in each group). Representative data of three similar experiments are shown. The results are expressed as mean ± s.d. Statistical analysis was performed according to the Tukey-Kramer test. *p<0.05, **p<0.01.

When the daily food intake per animal was compared in each group of mice by calculating Σ[(amounts given−amounts left)/total periods] throughout the feeding periods, CD1d^−/−^ female mice took in slightly smaller amounts of both SFD and HFD than the other two strains, WT and Jα18^−/−^ mice (Figure 2A). A similar tendency in food intake was observed in male mice (data not shown).

The BW of CD1d^−/−^ mice was significantly lower than that of WT and Jα18^−/−^ mice after 18 wk of HFD feeding (Figure 2B) (WT = Jα18^−/−^>CD1d^−/−^; *p*<0.01 between WT and CD1d^−/−^; Jα18^−/−^ and CD1d^−/−^). The difference in BW in each group of mice was attributable to the sum of the differences in weights of organs and tissues. Thus, the liver, perigonadal fat tissue (white adipose tissue; WAT) and fat tissue between the scapulae (brown adipose tissue; BAT) of each group were obtained and compared. Consistent with the differences in BW, CD1d^−/−^ mice had lower liver weight (Figure 2C) (WT = Jα18^−/−^>CD1d^−/−^; *p*<0.01 between WT and CD1d^−/−^; *p*<0.05 between Jα18^−/−^ and CD1d^−/−^), lower WAT weight (Figure 2D) (WT≥Jα18^−/−^>CD1d^−/−^; *p*<0.01 between WT and CD1d^−/−^; Jα18^−/−^ and CD1d^−/−^) and lower BAT weight (Figure 2E) (WT≥Jα18^−/−^>CD1d^−/−^; *p*<0.01 between WT and CD1d^−/−^), when compared to those of sex-matched WT or Jα18^−/−^ mice fed an HFD (closed bar). In SFD-fed mice, there were no significant differences in the weights of the whole BW, liver, WAT, and BAT among the three groups of mice (Figure 2B–E; open bar).

When serum lipids were quantified in the three groups of mice fed an HFD, WT and Jα18^−/−^ mice exhibited significantly higher levels of T-chol- and HDL-chol than CD1d^−/−^ mice (Figure S2A, B). The cholesterol level was consistent with the extent of adiposity, whereas the TG level was not different among the three groups (Figure S2C).

### HFD-induced hepatosteatosis and adipocyte hypertrophy are ameliorated in CD1d^−/−^ mice

**Figure 3:**
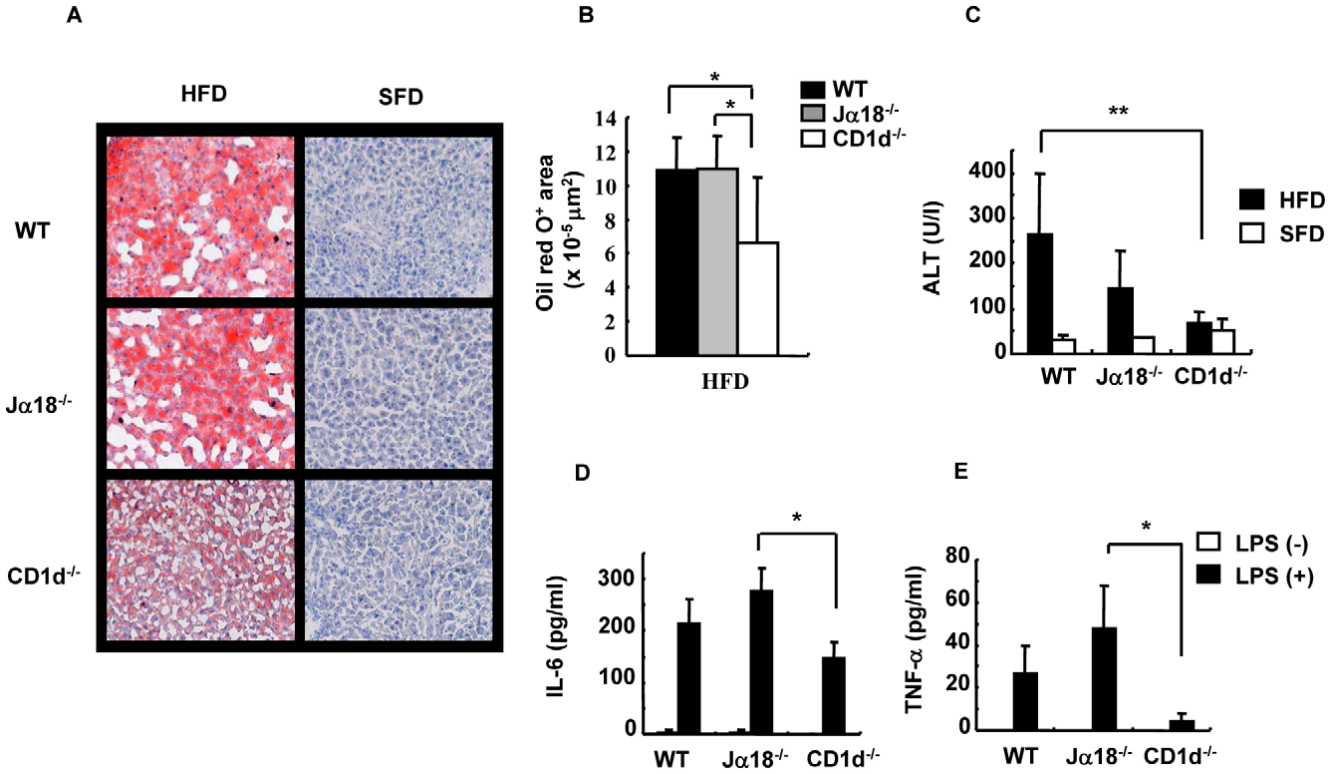
Histology and cytokine production in the liver. (A) A liver section was stained with ORO (frozen section) in SFD- and HFD-fed mice. (B) Red-stained lipid droplets in liver sections of HFD-fed mice were quantified with image analysis software. (C) Serum levels of ALT were quantified with the Drychem system. (D, E) The production of cytokines from HMNC of HFD-fed mice stimulated with LPS for 20 h (n = 3–4 female mice in each group). The results are expressed as mean ± s.d. Statistical analysis was performed according to the Tukey-Kramer test. *p<0.05, **p<0.01.

First, livers were histologically examined for major causes of weight gain among three strains of mice on HFD. WT and Jα18^−/−^mice on HFD had marked hepatosteatosis, which was revealed by red deposits on ORO staining (Figure 3A). On the other hand, livers of CD1d^−/−^ mice that appeared less fatty exhibited lighter deposits on ORO staining (Figure 3A). The ORO-positive regions in CD1d^−/−^ livers were significantly smaller than those of WT and Jα18^−/−^ mice (Figure 3B). The severity of steatosis appeared consistent with the liver weight and dyslipidemic level in each strain (Figure 2A and B). Furthermore, the ALT level, a marker of liver damage, was significantly suppressed in CD1d^−/−^ mice (Figure 3C). We also quantified pro-inflammatory cytokines that were secreted from HMNC in mice fed an HFD upon stimulation with LPS. Production of IL-6 and TNF-α in culture supernatants was increased in WT and Jα18^−/−^ mice, whereas it was suppressed in HMNC cultures of CD1d^−/−^ mice (Figure 3D, E). Thus, lipid-induced inflammation may be suppressed in the absence of CD1d and CD1d-restricted NKT cells.

**Figure 4:**
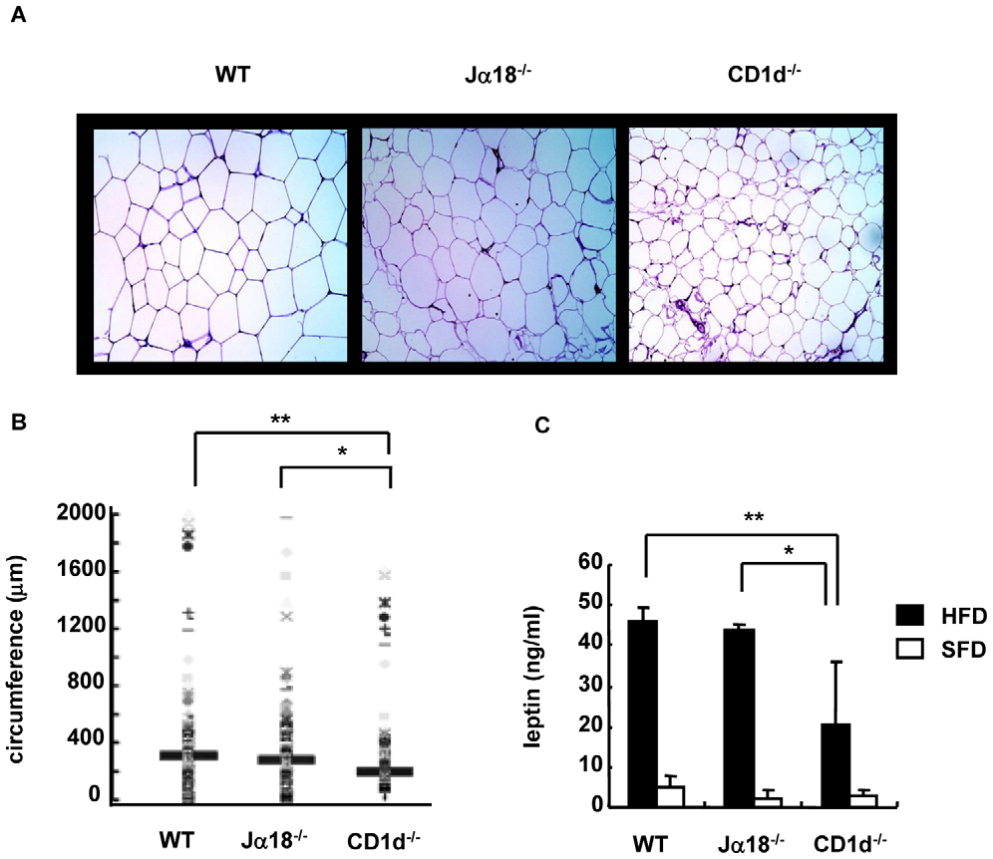
Histology of perigonadal adipose tissue and serum leptin level. (A, B) A paraffin section of perigonadal adipose tissue was stained with HE, and the circumference of adipocytes was morphometrically analyzed (10×10, 300–400 adipocytes measured) in HFD mice. (C) The serum leptin level was quantified by ELISA (n = 3–4 female mice in each group). Representative data of two similar experiments are shown. The results are expressed as mean ± s.d. Statistical analysis was performed according to the Tukey-Kramer test. *p<0.05, **p<0.01.

Next, we similarly analyzed adipose tissues that also contribute to the difference in BW in the three groups of mice. CD1d^−/−^ mice had apparently smaller adipocytes in adipose tissue even on HFD, whereas enlarged adipocytes occupied the entire tissue in WT and Jα18^−/−^ mice (Figure 4A). Of note, boundaries of adipocytes formed almost straight lines due to the close packing of adipocytes in WT mice (Figure 4A left panel). To compare adipocyte size in WAT of each group of mice, the circumference of each adipocyte was morphometrically compared (Figure 4B). The adipocytes from CD1d^−/−^ mice had significantly smaller circumference than those from WT and Jα18^−/−^ mice.

When leptin is overexpressed, mice demonstrate a lean phenotype [23]. To examine whether CD1d^−/−^ expressed a higher level of leptin in sera than WT and Jα18^−/−^ mice, serum leptin levels were quantified. However, CD1d^−/−^ showed the lowest level among the three strains of mice, which was proportional to the volume of WAT (Figure 4C).

### Analysis of infiltrated cellular components in liver of mice fed an HFD

**Figure 5:**
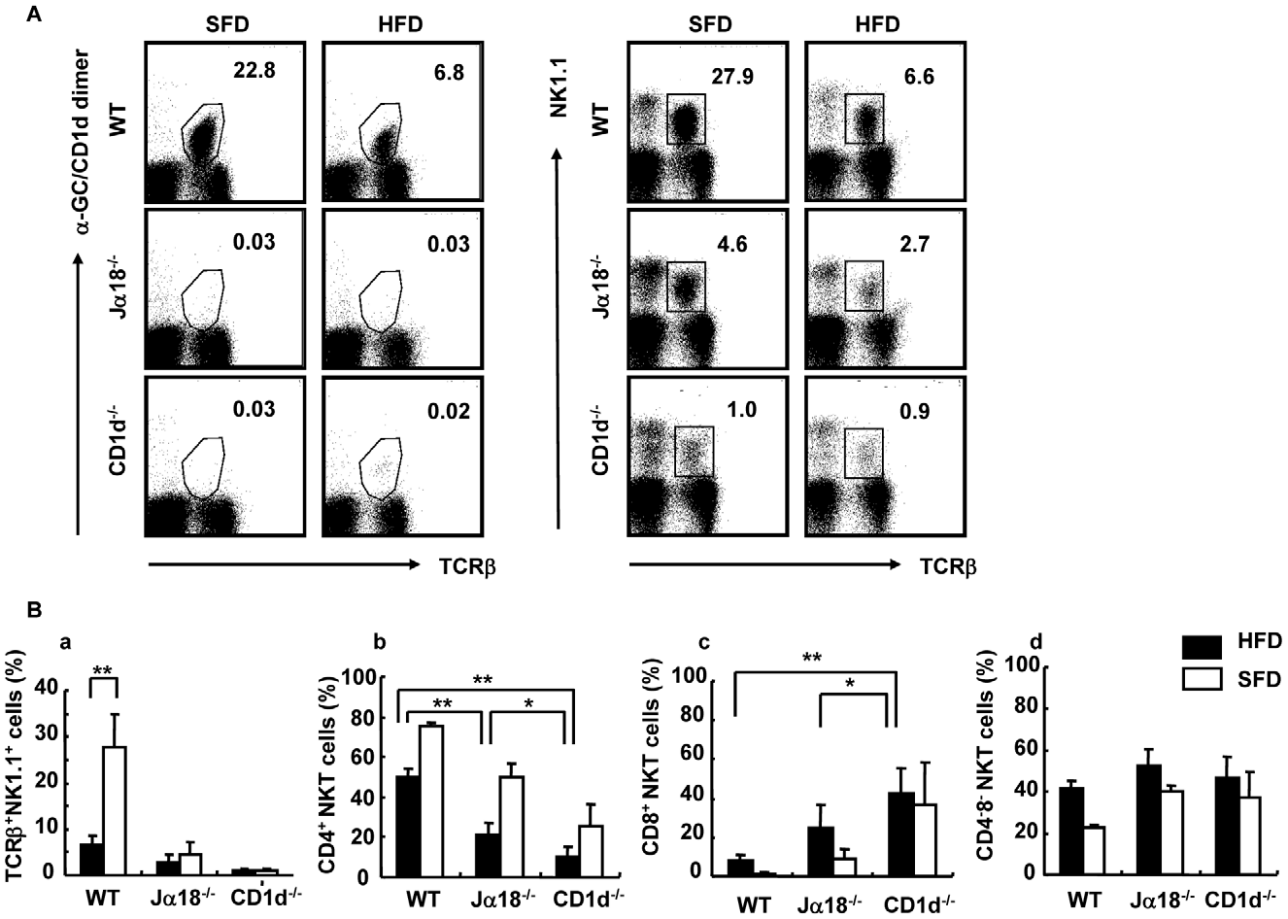
Flow cytometric analyses of HMNC in mice fed an SFD or an HFD. (A) A representative flow cytometric dot-plot defining the population of α-GalCer/CD1d dimer^+^TCRβ^+^ and NK1.1^+^TCRβ^+^ cells in the liver of SFD-fed mice (a, c) and HFD-fed mice (b, d). (B) The proportion of NK1.1^+^TCRβ^+^ cells (a), CD4^+^ (b), CD8^+^ (c), and CD4^−^CD8^−^ (d) NKT cells (n = 3–6 female mice in each group). Representative data of three similar experiments are shown. The results are expressed as mean ± s.d. Statistical analysis was performed according to the Tukey-Kramer test. *p<0.05, **p<0.01.

Our findings thus far showed that adiposity and hepatosteatosis were similar between WT and Jα18^−/−^ but were minimal in CD1d^−/−^ mice. The difference between Jα18^−/−^ and CD1d^−/−^ mice is solely the absence of type II NKT cells selected by CD1d in CD1d^−/−^ but not Jα18^−/−^ mice. To study the relationship between type I (iNKT) and type II NKT cells, we analyzed NKT cell subsets in liver and adipose tissue of each strain. As shown in our previous report with the short term feeding of HFD [18], the proportion of NKT cells in liver decreased in WT mice fed an HFD compared to mice on an SFD when the HMNC fraction was stained with a combination of either α-GalCer-loaded CD1d-dimer and anti-TCRβ mAb (iNKT cells) (Figure 5A-a, b; 22.8%−>6.8%) or anti-NK1.1 and TCRβ mAb (total NKT cells, including NKT-like cells) (Figure 5A-c, d; 27.9%−>6.6%). To detect the subset of type II NKT cells and NKT-like cells in the iNKT cell-deficient strains, the latter combination was employed. Although a significant decrease was demonstrated in NKT cells in WT mice (Figure 5B-a), there was no significant difference in the proportion of total NKT cells in Jα18^−/−^ and CD1d^−/−^ mice fed an HFD (Figure 5B-a), but Jα18^−/−^ mice appeared to exhibit a slight decrease in the prevalence of these cells on an HFD. To examine the residual NKT-like cells in the livers of Jα18^−/−^ and CD1d^−/−^ mice, staining with a combination of anti-NK1.1 and anti-TCRβ mAb was employed. As demonstrated in the FACS profiles, total NKT cells in WT mice were significantly decreased after HFD feeding (Figure 5B-a). As for the subset of residual NKT cells, the subset that expressed neither CD4 nor CD8 (CD4^−^8^−^ double negative; DN) did not differ regardless of the feeding regimen used or the mouse strains analyzed. The relative prevalence of the CD4^+^8^−^ subset was as follows: WT>Jα18^−/−^>CD1d^−/−^ mice, and the prevalence of the CD4^−^8^+^ subset had an inverse relationship with CD8^+^ cells (WT<Jα18^−/−^<CD1d^−/−^ mice) (Figure 5B-b, c). Although the total number of liver NKT cells was reduced, the residual population was mainly a CD4^+^ subset in WT mice and the remaining cells were a CD4^−^8^−^ subset (Figure 5B-d). The CD8^+^ subset was very minimal in WT mice. On the other hand, CD1d^−/−^ mice had residual numbers of hepatic NK1.1^+^TCRβ^+^, NKT-like cells, and about 40∼50% of the population was CD8^+^. There was no significant difference in the proportion of regulatory T (T_reg_) cells in HMNC among the three strains of mice (data not shown). It should be noted that NKT cells in the liver of WT mice fed with HFD were significantly reduced. This effect of lipid excess was evident as early as day 1 of HFD feeding (data not shown).

### Analysis of infiltrated cellular components in adipose tissue of mice fed an HFD

**Figure 6:**
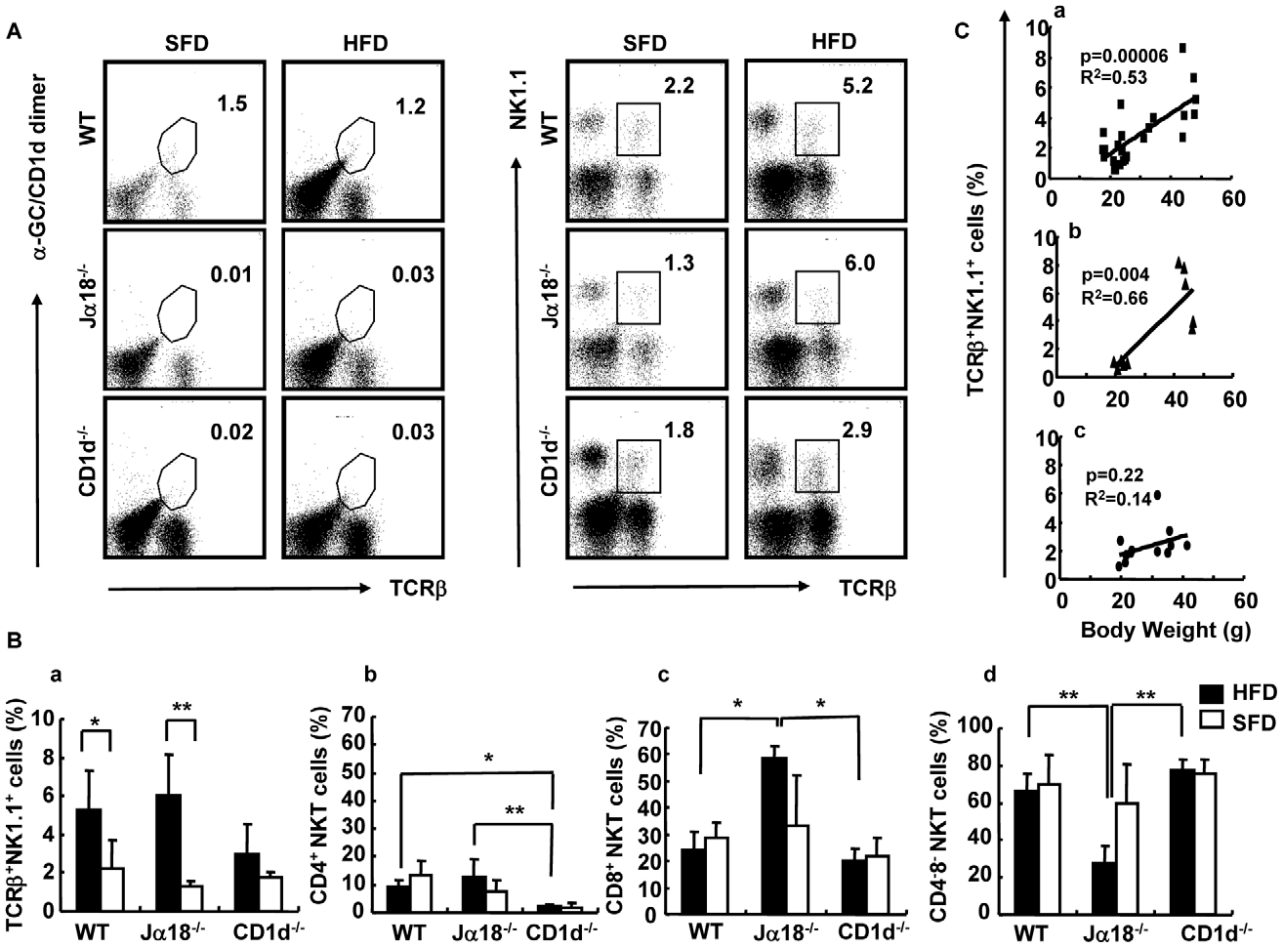
Flow cytometric analyses of NKT cells in adipose tissue in mice fed an SFD or an HFD. (A) A representative flow cytometric dot-plot defining the population of α-GalCer/CD1d dimer^+^TCRβ^+^ and NK1.1^+^TCRβ^+^ cells in adipose tissue of SFD-fed mice (a, c) and HFD-fed mice (b, d). (B) The proportion of each subset in SVF. NK1.1^+^TCRβ^+^ cells (a), CD4^+^ (b), CD8^+^ (c), and CD4^−^CD8^−^ (d) NKT cells (n = 3–6 female mice in each group). Representative data of three similar experiments are shown. The results are expressed as mean ± s.d. Statistical analysis was performed according to the Tukey-Kramer test. *p<0.05, **p<0.01. (C) The correlation between BW and the percentage of NKT cells in SVF by Pearson's correlation coefficient (R^2^) test. WT (a), *P* = 0.000006; Jα18^−/−^ (b), *P* = 0.004; and CD1d^−/−^ (c), *P* = 0.2. Values with *P<0 .05* were considered statistically significant.

The SVF in adipose tissue contains innate lymphocytes and Mφ even under normal conditions. In adipose tissue in obese mice, we readily detected increased numbers of mononuclear cells than in lean mice in spaces surrounded by adipocytes (data not shown). First, we analyzed NK1.1^+^TCRβ^+^ (NKT) cells and the CD4/8 subsets. Since the CD1d-restricted NKT cells are absent in CD1d^−/−^ mice, we used NK1.1^+^TCRβ^+^ staining to detect residual NKT-like cells instead of double stainig with a combination of α-GalCer-loaded CD1d-dimers and anti-TCRβ mAb. In WT mice, the proportion of iNKT cells in adipose tissue was not significantly different (Figure 6A-a, b; 1.5%−>1.2%), whereas there were significantly more NK1.1^+^TCRβ^+^ cells in mice fed an HFD compared to those on SFD, in the same experimental setting (Figure 6A-c, d; 2.2%−>5.2%). The percentage of NKT cells increased in WT and Jα18^−/−^ cells when the NKT cells of mice on HFD were compared with those on SFD (WT; *p*<0.05; Jα18^−/−^; *p*<0.01), whereas such an increase was not observed in CD1d^−/−^ mice (Figure 6B-a). Since CD1d^−/−^ mice lack CD4^+^ CD1d-restricted NKT cells, there were significant differences in the percentages (Figure 6B-b) (WT; *p*<0.05; Jα18^−/−^; *p*<0.01) and actual cell numbers (WT, Jα18^−/−^; *p*<0.05). Of note, the CD8^+^ subset was prominently increased in the adipose tissue of Jα18^−/−^ mice both in percentage (Figure 6B-c) and in number. The CD4^−^8^−^ subset of NKT cells appeared to be significantly decreased in Jα18^−/−^ mice fed an HFD, most likely due to the relative abundance of the CD4^−^8^+^ subset (Figure 6B-d). Similar results were obtained when analyzing actual cell numbers (WT>Jα18^−/−^>CD1d^−/−^) (data not shown).

We analyzed and compared the proportion of NKT cells at early and late phases (18-wk) of HFD feeding. In WT mice, the number of α-GalCer-CD1d-dimer^+^ cells and NK1.1^+^TCRβ^+^ cells in liver were increased at 1 wk of HFD-feeding, and numbers tended to decrease thereafter (Figure S3A). On the other hand, these cells, especially the NK1.1^+^TCRβ^+^ population, were gradually increased in adipose tissue (Figure S3B). The increase in NK1.1^+^TCRβ^+^ cells (percentage in SVF) in adipose tissue correlated with BW in WT and Jα18^−/−^ mice, whereas no correlation was found in CD1d^−/−^ mice (Figure 6C). A similar correlation was obtained between numbers of NK1.1^+^TCRβ^+^ cells (cell number/g adipose tissue) and BW (data not shown).

### Analysis of Mϕ in adipose tissue and cytokine production upon LPS stimulation

**Figure 7:**
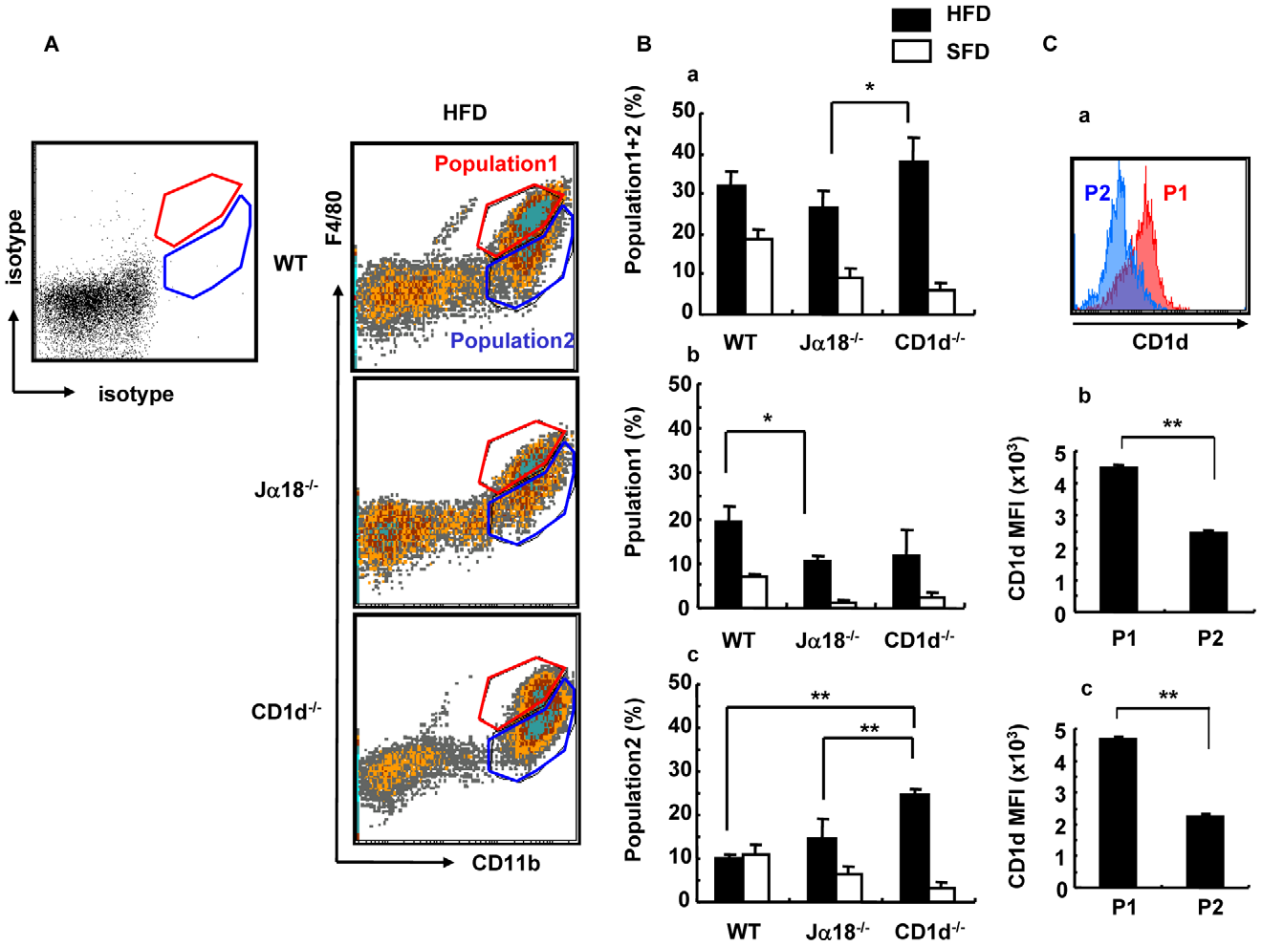
Flow cytometric analyses of infiltrated Mφ in perigonadal adipose tissue. (A) A representative flow cytometric dot-plot defining the F4/80^+^CD11b^+^ population in perigonadal adipose tissue in HFD-fed mice. F4/80^hi^ (Population 1; red gate) and F4/80^low∼−^ (Population 2; blue gate) are set to a staining with isotype control mAb. (B) The percentage of cells of CD11b^+^ (total) (a), Population 1 (b) and Population 2 (c). (C) CD1d expression by the Population 1 and Population 2 (a), MFI in WT (b) and Jα18^−/−^ (c) mice fed an HFD (n = 3–4 male mice in each group). The results are expressed as mean ± s.d. Statistical analysis was performed according to the Tukey-Kramer test. *p<0.05, **p<0.01.

Mϕ are another major cellular subset in SVF and may affect the metabolism of adipose tissue. Therefore, SVF preparations from the three strains of mouse were stained with F4/80 and CD11b (Figure 7A). The three strains had a similar pattern of F4/80^+^/CD11b^+^ staining. Although we anticipated that CD1d^−/−^ mice had fewer Mϕ than Jα18^−/−^ mice, the percentage of F4/80^+^/CD11b^+^ cells in CD1d^−/−^ mice was higher than that of Jα18^−/−^ mice (Figure 7B-a). However, when the F4/80^+^ population was divided into two subpopulations according to the MFI of F4/80 staining pattern, i. e., Population 1 (P1: F4/80^high^) and Population 2 (P2: F4/80^low∼−^) cells (both CD11b^+^), WT mice had higher frequencies of P1 than Jα18^−/−^ mice (Figure 7B-b), whereas CD1d^−/−^ mice had an increased frequency of P2 than WT and Jα18^−/−^ mice (Figure 7B-c). Analyses of actual cell numbers demonstrated the same tendency among the three mouse strains (data not shown). Of note, a higher MFI of CD1d expression was detected in the cells of the P1 population compared with the P2 population, both in WT and Jα18^−/−^ mice (Figure 7C-a, b, c).

**Figure 8:**
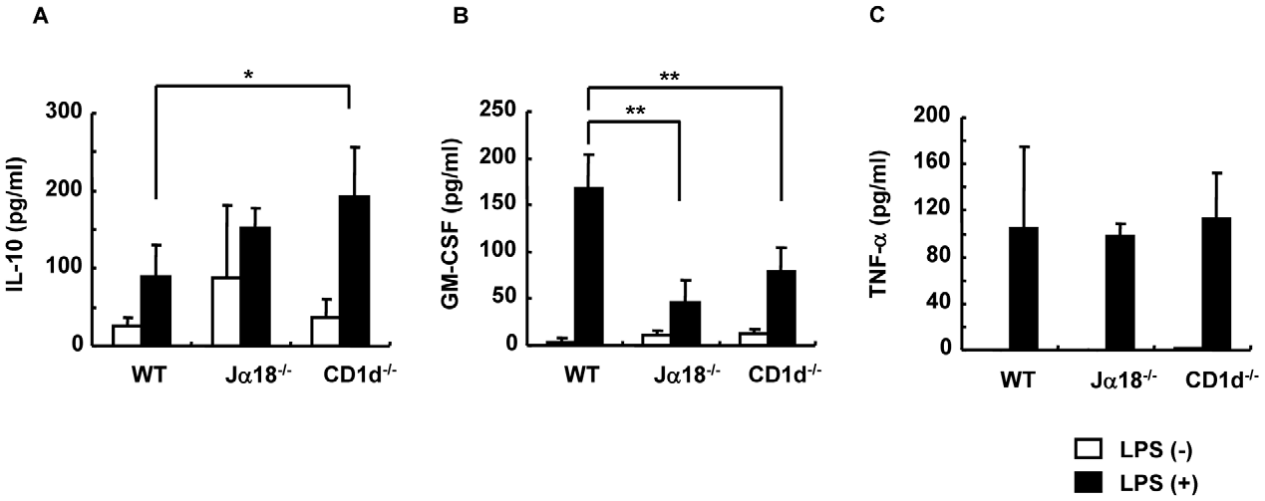
Cytokine production by Mϕ in perigonadal adipose tissue upon stimulation with LPS. (A–C) IL-10, GM-CSF and TNF-α concentrations after stimulation of Mϕ in perigonadal adipose tissue with LPS (1 µg/ml) for 20 h (n = 3–4 male mice in each group). Representative data of three similar experiments are shown. The results are expressed as mean ± s.d. Statistical analysis was performed according to the Tukey-Kramer test. *p<0.05, **p<0.01.

To examine functional differences in a specific Mφ population, total cells from the SVF fraction were stimulated with LPS for 20 h and cytokines were measured (Figure 8). IL-10 levels were significantly higher in SVF cultures from CD1d^−/−^ mice compared with WT mice (Figure 8A), and GM-CSF was significantly lower in Jα18^−/−^ and CD1d^−/−^ mice (Figure 8B). Notably, the production of TNF-α in the culture supernatant that could affect insulin resistance was not significantly different among the three strains (Figure 8C).

### Glucose and insulin tolerance are ameliorated CD1d^−/−^ mice

**Figure 9:**
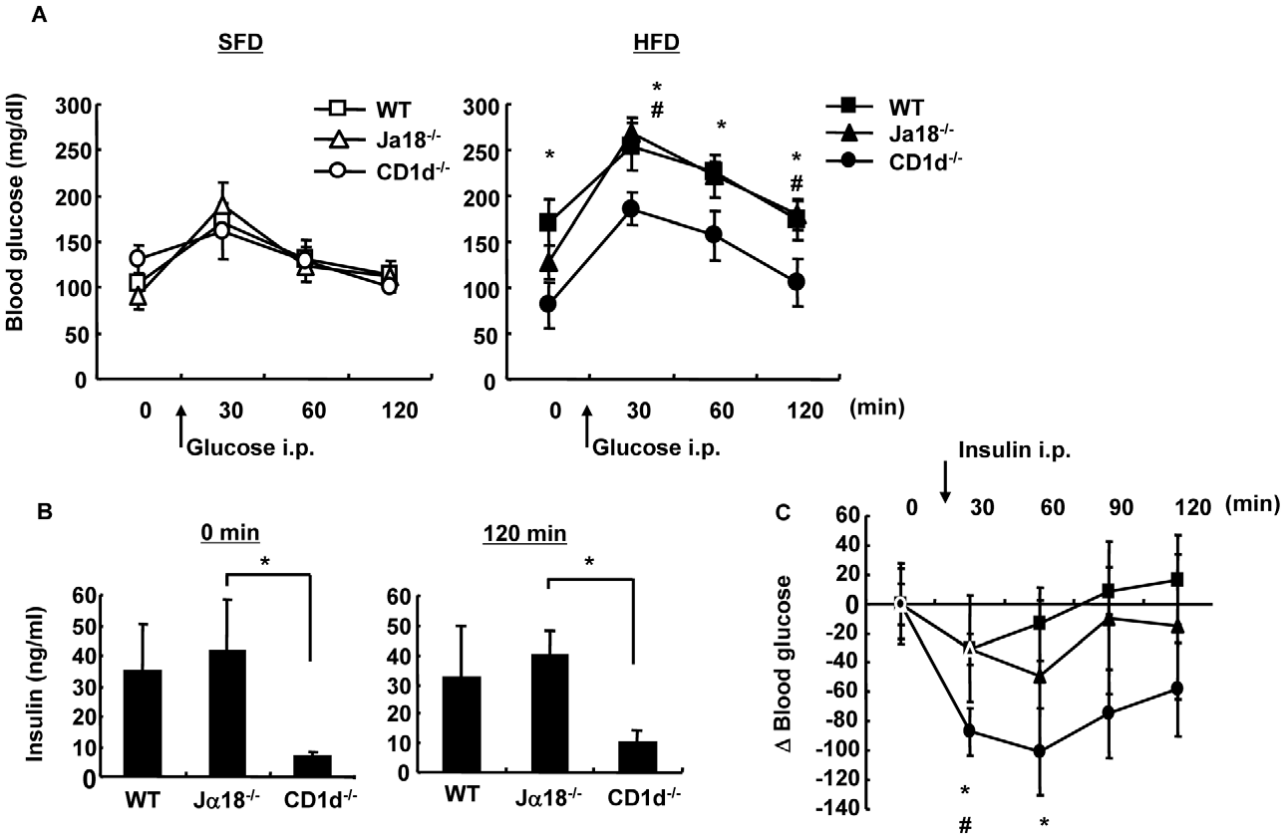
Glucose and insulin tolerance. (A) (IPGTT). Glucose (1 g/kg BW) was administered *i.p.* to female mice fed an SFD or an HFD. (B) The serum level of insulin 0 min and 120 min after glucose administration (*i.p.*) to HFD-fed mice was quantified by ELISA. (C) ITT. Insulin (0.75 U/kg BW) was administered *i.p.* to HFD-fed mice (n = 3–4 female mice in each group). Representative data of three similar experiments are shown. The results are expressed as mean ± s.d. Statistical analysis was performed according to the Tukey-Kramer test. *p<0.05 (WT versus CD1d^−/−^); ^#^p<0.05 (Jα18^−/−^ versus CD1d^−/−^).

Since the pattern of adiposity should reflect levels of glucose intolerance, IPGTT was performed in the three mouse strains (Figure 9). No difference was observed in fasting blood sugar levels and in elevation after *i.p.* administration of glucose over time in the three strains fed an SFD (Figure 9A, left). On the other hand, CD1d^−/−^ mice demonstrated the lowest fasting blood sugar level and a suppressed elevation profile after glucose administration among the three strains on an HFD (Figure 9A, right). Insulin levels were significantly higher in WT and Jα18^−/−^ mice before and at 120 min after infusion than that obtained in the respective groups on an SFD (data not shown) and for CD1d^−/−^ mice on an HFD, suggesting that insulin resistance was present in those strains (Figure 9B). To further examine insulin resistance, an ITT was performed in HFD-fed mice that had been fasted for 3.5 h [6] (Figure 9C). Following injection of insulin, blood glucose levels fell most prominently in CD1d^−/−^ mice at 30 and 60 min after the injection (Figure 9C). The decrease in glucose level in WT mice injected with α-GalCer was less than that of mice injected with vehicle (Figure S1B), suggesting that insulin resistance was reproduced in mice when iNKT cells were activated [18], although there was no net increase in BW with the treatment. Thus, CD1d^−/−^ mice developed the least resistance to insulin in the absence of both iNKT and type II NKT cells.

To examine whether type II NKT cells alone could reproduce the pathophysiological status observed in Jα18^−/−^ mice, HMNC obtained from either Jα18^−/−^ or CD1d^−/−^ mice were intravenously transferred to CD1d^−/−^ recipient mice and weight gain was examined on an HFD. CD1d^−/−^ mice that received HMNC from Jα18^−/−^ mice showed a slight increase in BW and exhibited a less profound decrease in blood glucose levels in the ITT (Figure S4A, C). Although no significant difference was detected with the IPGTT (Figure S4B), CD1d^−/−^ mice that received HMNC from Jα18^−/−^ mice showed higher insulin resistance than CD1d^−/−^ mice that received HMNC from CD1d^−/−^ mice. The percentage of NKT cells (NK1.1^+^ TCRβ^+^) and CD4^+^ NKT cells was similar in the liver and adipose tissue of CD1d^−/−^ hosts (Figure S4D-a, b). However, CD8^+^ NK1.1^+^ T cells in adipose tissue were significantly increased in mice transferred with HMNC from Jα18^−/−^-mice (Figure S4D-c), similarly to what we observed in Jα18^−/−^ mice fed an HFD.

## Discussion

Adipocytes, Mϕ and T cells are now thought to constitute a functional triad to promote obesity and obesity-associated disorders [1]–[3], [6]–[8]. Recently, the involvement of eosinophils and B cells has also been demonstrated [24], [25], implying that many other immune cells participate in the physiologic and pathologic processes of adipocyte hypertrophy. This may be a natural process since adipose tissue is thought to be an ancestor of lymphoid organs [5], [14].

In the present study, we demonstrated that an innate lymphocyte of the T cell lineage, the CD1d-restricted NKT cell, also has an active role in the development of obesity. When fed an HFD (CLEA Japan), CD1d^−/−^ mice gained the least weight compared to Jα18^−/−^ and WT mice. This difference was demonstrated when mainstream CD8^+^ T cells were primarily unaffected. Furthermore, similar results were obtained with different types of HFD (Harlan Teklad [26] and our own original preparation [18]), suggesting that the suppression of weight gain was solely dependent on the deficiency of CD1d-restricted NKT cells but not on the specific source of fat, i.e. fatty acid composition.

Since Jα18^−/−^ mice gained comparable BW as WT mice, the residual CD1d-restricted, type II NKT cells appeared to contribute to the accumulation of visceral fat in the body. In HFD-fed WT and Jα18^−/−^ mice, the number of NK1.1^+^TCRβ^+^ cells increased along with the hypertrophic change of abdominal WAT (Figure 4, 6), whereas the α-GalCer-CD1d dimer^+^ population did not change in adipose tissue of WT mice, suggesting that the type II NKT cells were the major population affecting adipose tissue metabolism. Accordingly, the transfer of NKT cells from Jα18^−/−^ mice to CD1d^−/−^ mice resulted in weight gain and significant increase in insulin resistance (Figure S4). Both the percentage and the cell number of NK1.1^+^TCRβ^+^ cells in adipose tissue correlated with the BW weights in Jα18^−/−^ as well as in WT mice, whereas no correlation was found in CD1d^−/−^ mice (Figure 6C). When the NK1.1^+^TCRβ^+^ cells in adipose tissue were analyzed, we were unable to detect these cells with sulfatide-loaded CD1d-multimers that are specific for a subset of type II NKT cells [27] (data not shown). Of note, these cells exhibited a biased repertoire in specific Vβs in HFD-fed WT mice (data not shown), although the significance of this finding remains unclear. It should also be noted that α-GalCer administration did not boost weight gain in WT mice fed an HFD, although insulin action was blunted with α-GalCer administration. This result indicated that insulin resistance but not adiposity could be reproduced through the activation of type I NKT cells (Figure S1A).

Nishimura et al demonstrated that CD8^+^ T cells are important for recruitment of Mφ and chronic inflammation in adipose tissue, which was proposed to propagate inflammation [6]. In our experiments, the CD8^+^ subset of NK1.1^+^ T cells, a rather rare subset [28], increased in adipose tissue in WT and Jα18^−/−^ mice. Although we have not analyzed TCR Vβ chain usage, Nishimura described them as Vβ7 and Vβ10b [6]. Since Vβ7 is one of the preferred Vβ chains of NKT cells [29], it is possible that these cells or a portion of these cells include NKT cells. Very recently, Mantell et al. reported that no protection against metabolic abnormalities was noted in CD1d^−/−^ mice fed an HFD [30]. They demonstrated no significant differences between WT and CD1d^−/−^ mice in physiologic, metabolic, and inflammatory parameters. Nevertheless, since there was an early and reversible decrease in liver NKT cells, mice appeared to respond to an HFD. Specific reasons for these divergent findings will need to be explored in future studies. One possibility might be the colonization of mice from different animal facilities with different flora. An important role of intestinal flora in the process of obesity has emerged [31], and the differential dominance of certain species of microbes has been demonstrated in obese or anorexic human individuals [32]. Furthermore, in toll-like receptor 5 knockout (TLR5^−/−^) mice, metabolic syndrome was induced by altered gut microbiota with hyperphagic traits [33]. Moreover, these metabolic changes could be conferred to WT, germ-free mice upon transfer of the gut microbiota derived from TLR5^−/−^ mice. Although adaptive immunity is not directly related to the process of obesity in TLR5^−/−^ mice, it should be noted that the development of adiposity could be controlled by balancing the complex microbiota. Since bacterial colonization in the intestine is regulated by a CD1d-dependent, NKT cell-mediated mechanism [34], it is possible that NKT cell deficiency may alter the intestinal flora, and thus influence either an adipogenic or anti-adipogenic host milieu.

The recruitment of Mφ towards visceral fat, also known as adipose tissue Mφ (ATMφ; CD11b^+^F4/80^+^), has also been thought to be a hallmark of obesity [1], [2]. The F4/80^+^ fraction can be subdivided into the F4/80^high^ and F4/80^low^ populations [35]. In the present study, ATMφ of WT and Jα18^−/−^ mice had an increased F4/80^high^ population, whereas CD1d^−/−^ mice predominantly had an increased percentage and number of the F4/80^low^ population. The F4/80^high^ population exhibits higher expression of TLR-4, TNF-α, and PPARγ than the F4/80^low^ population. Therefore, this subset represents pro-inflammatory Mφ (M1) and accumulates in adipose tissue of obese mice [36]. On the other hand, the F4/80^low^ population exhibits higher IL-4 expression and represents anti-inflammatory Mφ [36]. Furthermore, elimination of the F4/80^high^ population is associated with improved insulin sensitivity [37]. Our studies suggest that the predominance of F4/80^high^ ATMφ is likely dependent on the presence of type II NKT cells, since it was observed in WT and Jα18^−/−^ mice. Consistently, SVF that contained more F4/80^low^ Mφ from CD1d^−/−^ mice produced more IL-10 upon stimulation with LPS (Figure 8). The anti-inflammatory milieu induced by Mφ polarization may ameliorate the development of obesity in CD1d^−/−^ mice. It should also be noted that F4/80^high^ ATMφ expressed higher levels of CD1d than F4/80^low^ Mφ in WT and Jα18^−/−^ mice, suggesting that NKT cells might better polarize these cells to cause adipose tissue inflammation. Alternatively, F4/80^high^ ATMφ might express higher levels of CD1d as a result of the interaction with type II NKT cells.

The present study suggests that type II NKT cells may serve as a potential target for controlling the volume of visceral fat and insulin resistance. A possible approach may be the administration of a CD1d-binding ligand to modulate NKT cell function. Indeed, Ilan's group has demonstrated that the administration of glucocerebroside (β-glucosylceramide; β-GlcCer) or the direct transfer of NKT cells ameliorated metabolic aberration and steatohepatitis of leptin-deficient *ob/ob* mice [38], [39]. They demonstrated that the therapeutic effects of β-GlcCer were associated with a Th2-shift (decreased IFN-γ and increased IL-10) and the redistribution of NKT cells in the liver to the periphery. Furthermore, β-GlcCer modulated the lipid rafts of NKT cells, thus altering the expression of molecules of cellular signaling, such as flotillin-2, Lck, and STAT1 [40]. Although the mode of action appears to be dependent on iNKT cells, it remains possible that these CD1d-binding non-stimulatory ligands may simply function as antagonists for all CD1d-restricted NKT cells.

Of special interest, Lynch et al. demonstrated that NKT cells and CD1d^+^ cells are abundantly detected in the omentum tissue in humans [41]. Half of the NKT (CD3^+^CD56^+^) cells stained with the 6B11 mAb [42], indicating that they were iNKT cells, but the remaining cells likely are type II NKT cells. They also reported a net decrease of iNKT cells in severely obese individuals with a relative increase of CD56^+^ T cells (healthy, 49.9%; obese, 61.9%) and discussed that the relative immunocompromised status in obesity due to the decreases in iNKT cells might be related to sensitivity to infections [40]. On the other hand, the omentum may be a source of not only iNKT cells but also type II NKT cells that are capable of inducing adiposity in the abdominal cavity.

In conclusion, we showed that deficiency of CD1d-resricted NKT cells suppressed the development of obesity, likely because inflammation of adipose tissue and liver were ameliorated and reduced levels of insulin resistance were induced. NKT cells, especially the type II subset, may be the initial subset of T cells that recognizes and responds to lipid excess. Thus, CD1d-restricted, type II NKT cells may be a novel target for therapeutic intervention in the metabolic syndrome. Searching for regulatory ligands and novel ways of regulation that include the selective elimination of pathogenic type II NKT cells might permit the control of not only metabolic syndrome but also various diseases related to lipid inflammation mediated by NKT cells [43].

## Supporting Information

Figure S1 **BW and insulin resistance of mice administered α-GalCer.** (A) BW was determined weekly in WT mice given either vehicle or α-GalCer (0.1 µg/g BW). (B) ITT was performed for WT mice injected with vehicle or α-GalCer (n = 5 female mice in each group). Representative data of two similar experiments are shown. The results are expressed as mean ± s.d. Statistical analysis was performed according to the Student's *t*-test. *p<0.05.
(TIFF)

Figure S2 **Serum T-chol-, HDL-chol, and TG levels.** (A–C) Serum T-chol-, HDL-chol, and TG level after an 18 wk feeding and a 16 h fasting period (n = 6–8 female mice in each group). Representative data of three similar experiments are shown. The results are expressed as mean ± s.d. Statistical analysis was performed according to the Tukey-Kramer test. *p<0.05, **p<0.01.
(TIFF)

Figure S3 **Dynamics of NKT cells in liver and adipose tissue during the early phase of feeding.** The cell number of iNKT cells and NK1.1^+^TCRβ^+^ cells in liver (A) and in adipose tissue (B) at 1, 2, 3, and 18 wk of HFD-feeding (n = 3–6 female mice in each group). Representative data of two similar experiments are shown. The results are expressed as mean ± s.d.
(TIFF)

Figure S4 **Effects of adoptive transfer of Jα18^−/−^ HMNC to CD1d^−/−^ mice.** (A) CD1d^−/−^ mice received HMNC (1×10^6^) from Jα18^−/−^ mice since 8 wk of age on an HFD. BW was determined weekly. (B, C) IPGTT and ITT were performed at 14 wk of HFD feeding. (D) The number of NK1.1^+^TCRβ^+^ cells (a) and the CD4/8 proportion in liver and adipose tissue (b, c) were analyzed by flow cytometry (n = 5 male mice in each group). Representative data of two similar experiments are shown. The results are expressed as mean ± s.d. Statistical analysis was performed according to Student's *t*-test. *p<0.05.
(TIFF)
