# Supplementary figures and images for: Type II NKT Cells Stimulate Diet-Induced Obesity by Mediating Adipose Tissue Inflammation, Steatohepatitis and Insulin Resistance
Source: PLoS One. 2012 Feb 22;7(2):e30568. doi: 10.1371/journal.pone.0030568 (PMC3284453; doi:10.1371/journal.pone.0030568)

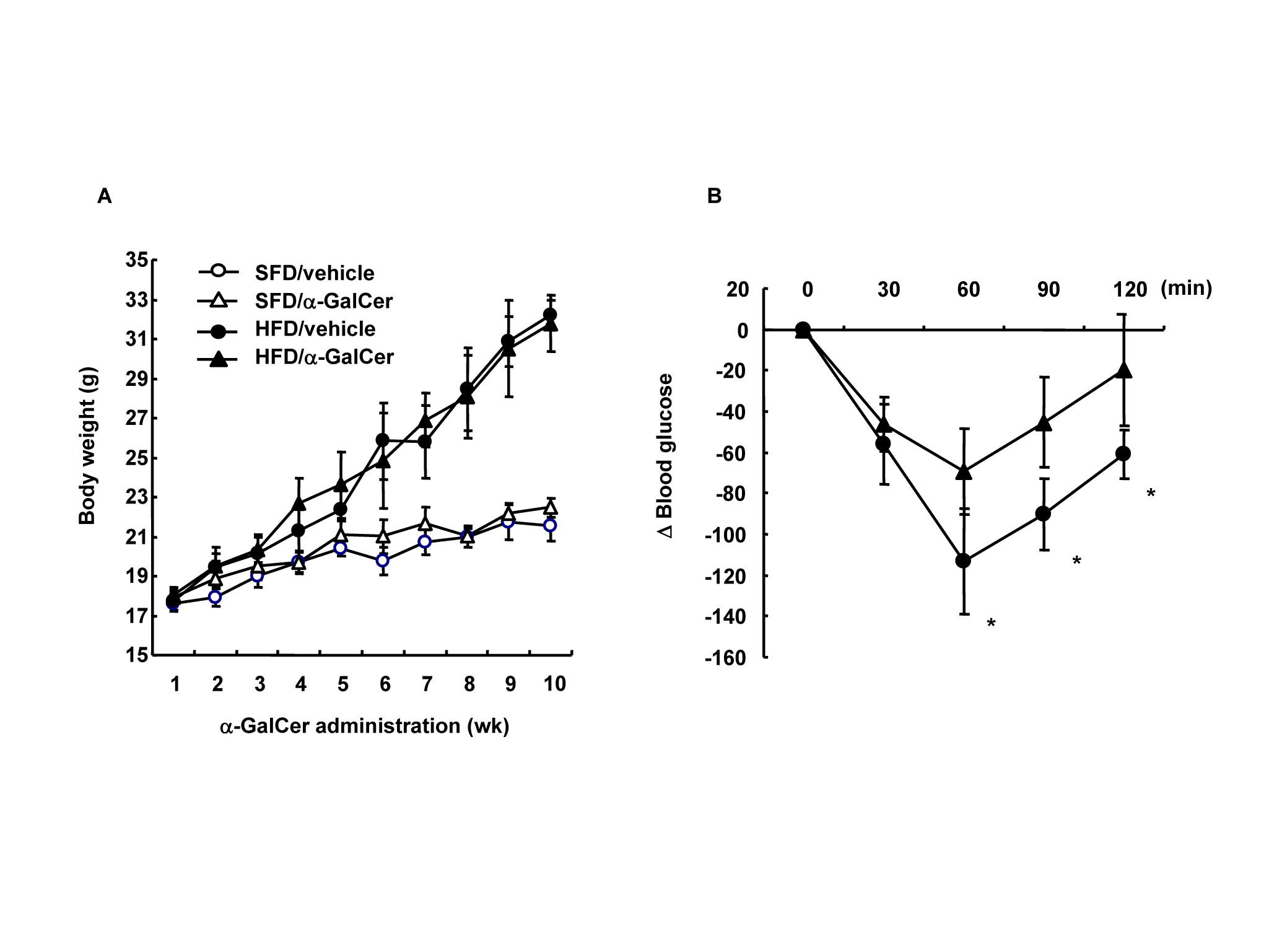

Supplement: Figure S1 — BW and insulin resistance of mice administered α-GalCer. (A) BW was determined weekly in WT mice given either vehicle or α-GalCer (0.1 µg/g BW). (B) ITT was performed for WT mice injected with vehicle or α-GalCer (n = 5 female mice in each group). Representative data of two similar experiments are shown. The results are expressed as mean ± s.d. Statistical analysis was performed according to the Student's t-test. *p<0.05. (TIFF) [file pone.0030568.s001.tiff]

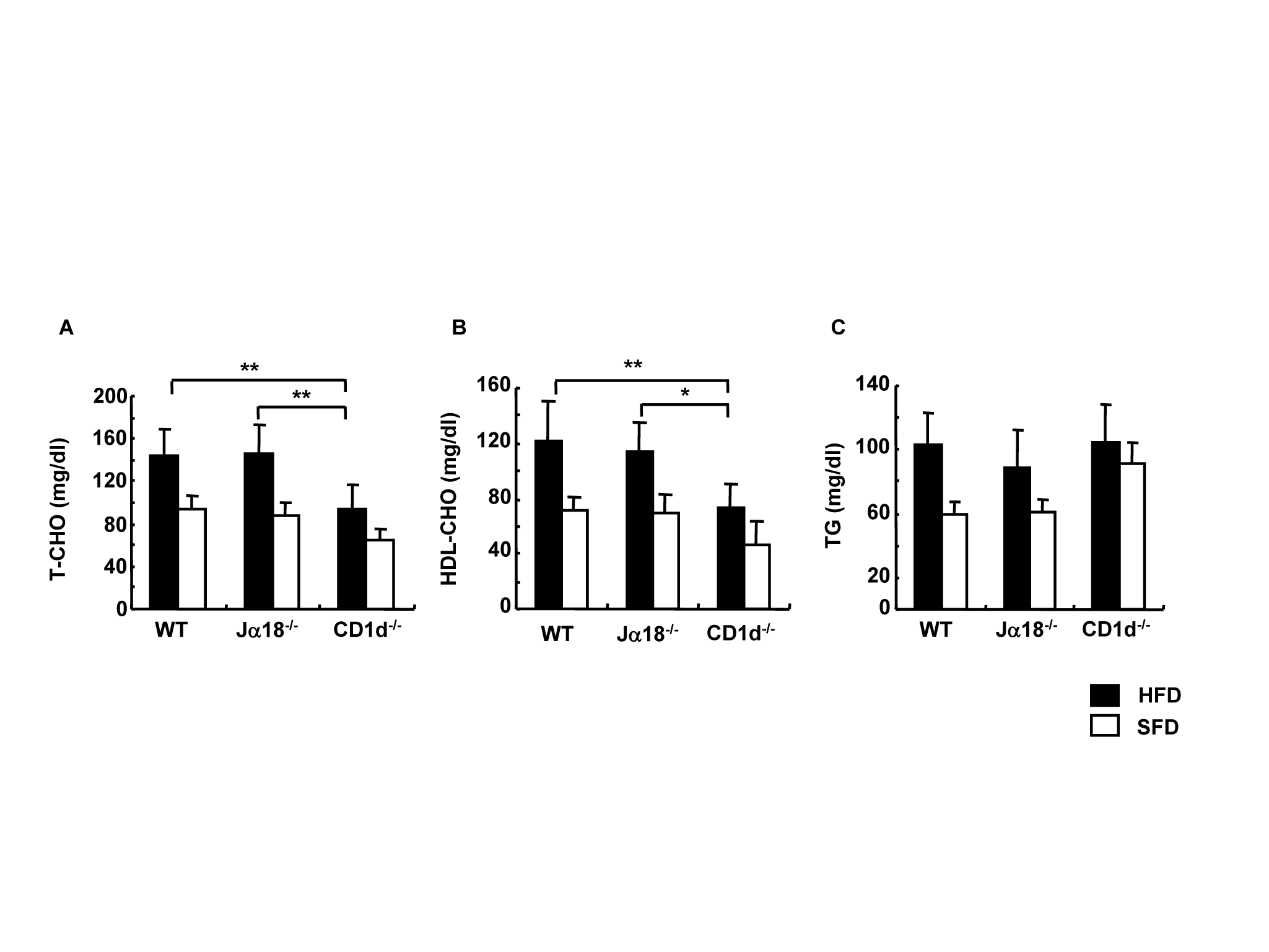

Supplement: Figure S2 — Serum T-chol-, HDL-chol, and TG levels. (A–C) Serum T-chol-, HDL-chol, and TG level after an 18 wk feeding and a 16 h fasting period (n = 6–8 female mice in each group). Representative data of three similar experiments are shown. The results are expressed as mean ± s.d. Statistical analysis was performed according to the Tukey-Kramer test. *p<0.05, **p<0.01. (TIFF) [file pone.0030568.s002.tiff]

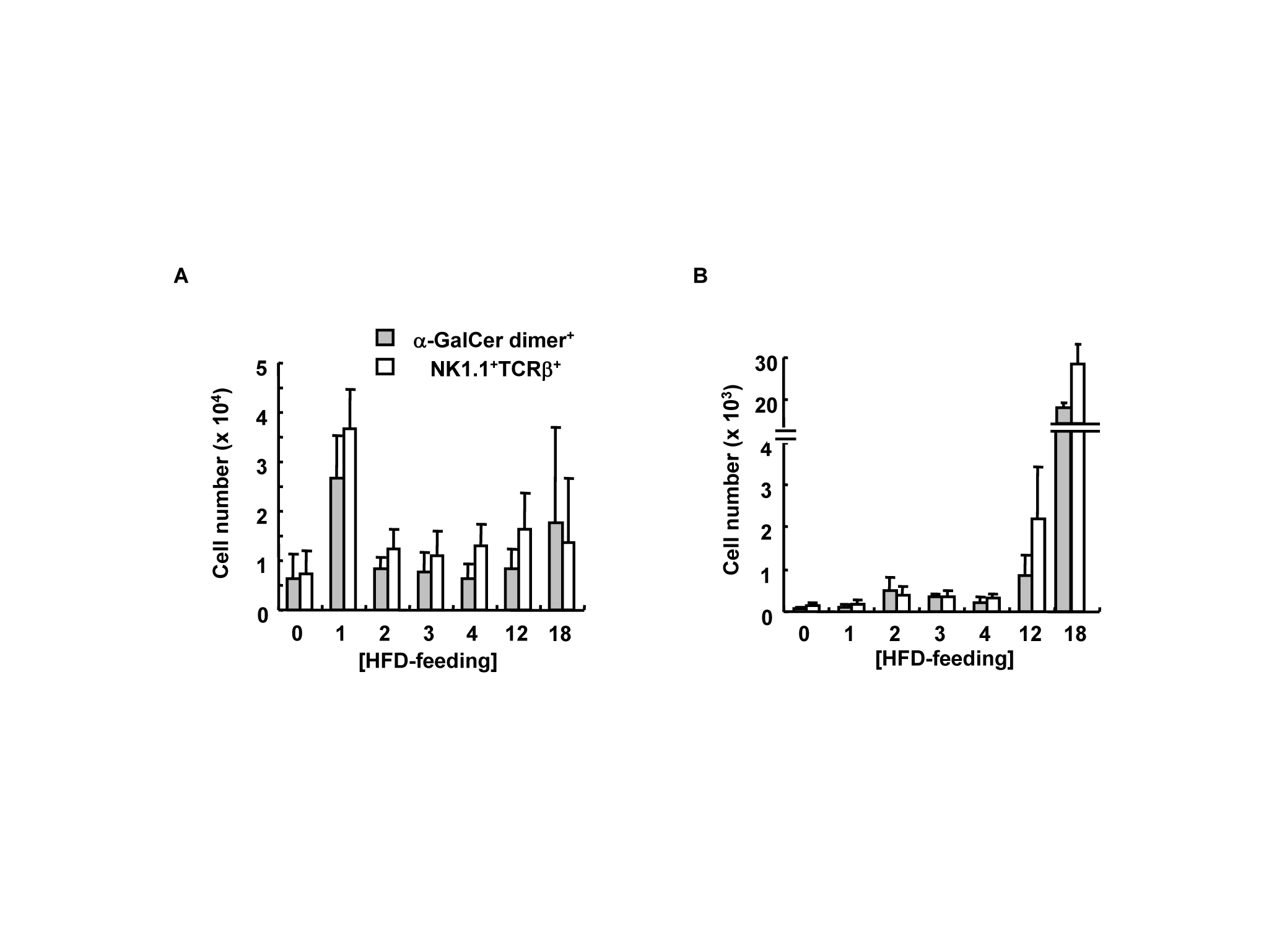

Supplement: Figure S3 — Dynamics of NKT cells in liver and adipose tissue during the early phase of feeding. The cell number of iNKT cells and NK1.1+TCRβ+ cells in liver (A) and in adipose tissue (B) at 1, 2, 3, and 18 wk of HFD-feeding (n = 3–6 female mice in each group). Representative data of two similar experiments are shown. The results are expressed as mean ± s.d. (TIFF) [file pone.0030568.s003.tiff]

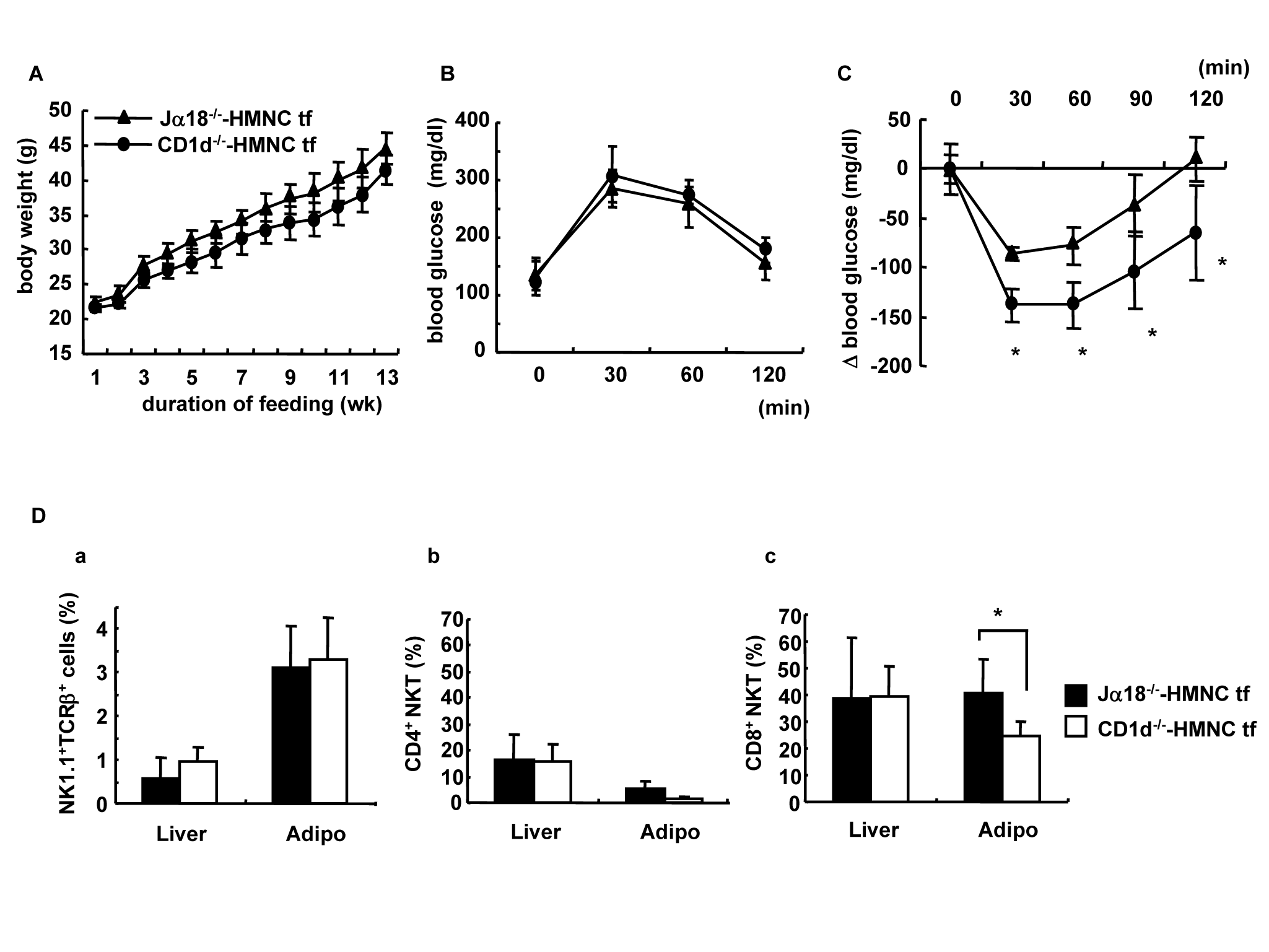

Supplement: Figure S4 — Effects of adoptive transfer of Jα18−/− HMNC to CD1d−/− mice. (A) CD1d−/− mice received HMNC (1×10^6^) from Jα18−/− mice since 8 wk of age on an HFD. BW was determined weekly. (B, C) IPGTT and ITT were performed at 14 wk of HFD feeding. (D) The number of NK1.1+TCRβ+ cells (a) and the CD4/8 proportion in liver and adipose tissue (b, c) were analyzed by flow cytometry (n = 5 male mice in each group). Representative data of two similar experiments are shown. The results are expressed as mean ± s.d. Statistical analysis was performed according to Student's t-test. *p<0.05. (TIFF) [file pone.0030568.s004.tiff]
